# An Analysis of Computed Tomography Imaging Features and Predictive Factors for Postoperative Recurrence and Metastasis of Abdominal Paragangliomas

**DOI:** 10.1155/2022/8638588

**Published:** 2022-02-21

**Authors:** Bailing Dai, Jie He, Xiandi Zhu, Zongyu Xie, Cui Zhang, Xiaoli Zhou, Zhao Yang, Jian Wang

**Affiliations:** ^1^Department of Radiology, Tongde Hospital of Zhejiang Province, Hangzhou, Zhejiang, China; ^2^Department of Radiology, Sir Run Run Shaw Hospital, Zhejiang University School of Medicine, Hangzhou, Zhejiang, China; ^3^Department of Radiology, The First Affiliated Hospital of Bengbu Medical College, Bengbu, Anhui, China

## Abstract

**Methods:**

We studied 51 abdominal PGL patients at the First Affiliated Hospital of Bengbu Medical College, Tongde Hospital, and Sir Run Shaw Hospital, Hangzhou, Zhejiang Province, China, from June 2009 to May 2019. Thereafter, the clinical research data, tumor biomarkers, and CT features were compared between the aggressive PGLs and the nonaggressive PGLs using independent-samples *t*-tests and chi-square tests.

**Results:**

Of the 51 cases, 43 were benign and 8 had malignant tendencies. Postoperative recurrence and metastasis were more likely to occur when the tumor diameter was >8 cm or/and the enhancement degree was not obvious. Clinical symptoms, tumor markers, sex, age, and CT image characteristics including morphology, presence of cystic degeneration, “pointed peach” sign, calcification, hemorrhage, enlarged lymph nodes, and peritumor and intratumor blood vessels were not significantly different between the two groups (*p* > 0.05).

**Conclusion:**

Our findings suggest that CT features, including size >8 cm and enhancement degree, could provide important evidence to assess risk factors for aggressive PGLs.

## 1. Introduction

Pheochromocytomas (PCC) and extra-adrenal paragangliomas (PGLs) are identical diseases that are named differently depending on their anatomical location. The World Health Organization decided to assign the term “pheochromocytoma” to refer exclusively to tumors occurring in the adrenal medulla and “extra-adrenal PGLs” as tumors occurring in other tissues [1]. PGL is a rare neuroendocrine neoplasm observed in patients of all ages, with an estimated incidence of 3/1,000,000 [2]. Abdominal PGLs can be located in the organ of Zuckerkandl, which lies anteriorly to the aortic bifurcation, but they can also be found in paraaortic locations, in the bladder base, or even in the pancreas, liver, and mesentery [3]. A majority of patients have nonspecific clinical symptoms, including slight abdominal discomfort or pain, headaches, dizziness, high blood pressure, palpitations, dysuria, or nonspecific clinical signs, such as the presence of an abdominal mass, while several patients present diarrhea, vomiting, nausea, and menstrual disorders [4]. Since all patients with PGLs have a risk of recurrence and primary tumor resection does not completely eliminate the risk of tumor persistence and recurrence, they should be followed up for at least 10 years for recurrent disease and new tumor formation [5]. Accurate prediction of the prognostic risk factors of PGLs preoperatively and developing a well-grounded therapy guide are determinative to increasing the survival rate. Clinically, several biomarkers, including cortisol, CEA, CA199, CA125, and renin, are used to be comprehensive tumor diagnosis. However, there are currently no diagnostic biomarkers for malignant PGLs. Histologically, there are no reliable markers of malignancy. Existing pathological grading systems for determining whether PGLs are “malignant” or have “metastatic potential” have limited predictive strength [6, 7].

Computed tomography (CT) is currently identified as the preferred imaging method for the initial evaluation and follow-up of patients with PGLs. Little is known about the CT manifestations of abdominal PGLs owning to their rarity [8]. Only sporadic case reports or small-sample-size studies of patients have been reported [9–11]. Reports on the CT features of retroperitoneal PGLs showed variation over a wide range, and it was confusing to differentiate PGLs from retroperitoneal neoplasms, including stromal tumors, nerve sheath tumors, and hyaline vascular Castleman disease. The previous reviews [12–15] concluded that CT imaging characteristics, including tumor margin, tumor size, tumor shape, direct organ invasion, necrosis, calcification, presence of enlarged feeding or draining vessels within the mass, lymphadenopathy, and enhancement mode, were shown to be significant risk factors for malignant PGLs.

Therefore, in this study, we assessed the differences in clinical data and tumor markers between the aggressive and nonaggressive PGLs. Moreover, we attempted to clarify whether there are representative CT image features that assist in prognostic and predictive assessment for PGLs.

## 2. Materials and Methods

### 2.1. Patient Population

This was a multicenter retrospective cohort study. We investigated the histopathology data base on our institution for all cases of paragangliomas confirmed histologically. The records included cases from the First Affiliated Hospital of Bengbu Medical College, Tongde Hospital of Zhejiang Province, and Sir Run Shaw Hospital, Hangzhou, Zhejiang Province, China, from June 2009 to May 2019. A total of 51 patients were enrolled in this study with 8 PGL patients with metastasis or recurrence composed the aggressive group, while the nonaggressive group was composed of 43 PGL patients without recurrence or metastasis. This retrospective observational study was obtained for institutional review board approval, and all participants provided written informed consent. We joined in patients who fulfilled the following inclusion criteria: (1) contrast-enhanced CT images and clinical information were available; (2) the surgery was performed after the CT examination; (3) the histopathological finding was proved to be PGL.

### 2.2. Data Collection

Clinical data, including the patient's case number, medical history, clinical symptoms, tumor markers, age, and sex, were collected retrospectively from the patients' medical records.

### 2.3. Imaging Examination Methods

#### 2.3.1. CT Examination

All of the CT images were acquired from one of the following three multidetector CT scanners: a 64-multidetector-row CT scanner (Siemens Definition AS or SOMATOM Definition Flash; a Light Speed VCT (GE Healthcare, Milwaukee, WI, USA) or Siemens Healthcare, Erlangen, Germany) was used. Patients were required not to eat or drink anything for 4–6 h before the examination. The scanning parameters included 120 kv tube voltage, 150–250 mA tube current, 64 × 0.625 mm detector collimation, 350 × 350 mm field of view, 5 mm section thickness, and 1–1.5 mm reconstruction interval. After finishing the unenhanced CT, a peripheral intravenous dose of 120 ml of nonionic iodinated contrast agent (dose 1.8 mL/kg, injection rate 2.5–3.0 mL/s) was given to the patients at the rate of 3–4 ml/s. An axial CT scan of the arterial and venous phases was initiated 25–30 s and 65–80 s after the contrast agent injection, respectively. All of the CT images were collected during an inspiratory breath hold.

### 2.4. CT Image Analysis

The abdominal CT scans images of the 51 patients were assessed by two experienced radiologists with 5 and 8 years' work experience, who were blinded to the pathologic findings. Lesion location, size (long diameter, short diameter), shape, boundary, uniformity, presence or absence of calcifications, cystic degeneration/necrosis, the number of cystic degeneration (single or multiple), enlarged lymph nodes, hemorrhage, and peritumor and intratumor enlarged vessels feeding or draining the mass were recorded. The size of the mass was measured using the maximum diameter line. Plain scan density of the lesions (CTU), the CT value of enhanced scan arterial and portal venous (CTA, CTV), net value of enhancement at the arterial (DEAP) and venous stages (DEPP) (formula = enhancement CT value − plain CT scan value), and enhancement degree (ED)were recorded. The area of interest (ROI) was drawn on the lesion parenchyma of the two groups, and the CT value at each stage was measured. During the measurement, it was ensured that the ROI was as large as possible to cover all solid components and there were no blood vessels, necrotic tissues, or calcifications in view. The CT values of 3–5 ROIs in the lesion area were measured, and the average CT value was calculated. The different ROI locations and the sizes of the same case were similar. They were divided into 3 groups based on the enhancement degree, the CT enhancement degree value (<40 HU) was defined as mild or moderate enhancement degree; the CT enhancement degree value (>60 HU) was defined as significant enhancement; the CT enhancement degree value of the remaining group ranges from 40 HU to 60 HU.

### 2.5. Immunohistochemistry

Surgical information was described from the author's first-hand experience and the retrospective analysis of the operative documentation as well as the photographic evidence; it included the presurgical differential diagnosis, type of procedure, and intraoperative findings. Histological characteristics were collected from formal histopathology reports both macroscopically (shape, color, consistency, and encapsulation) and microscopically (cellular arrangement, necrosis, immunostaining for neuron-specific enolase, chromogranin-A, synaptophysin, S100, GFAP, and additional markers where available).

### 2.6. Follow-Up

After surgery 10–14 days, patients were followed up to determine whether the tumor remained; all of PGL patients should be followed up every six months, and the follow-up period was 120 months. The follow-up rate was 100%. All patients were followed up with regard to postoperative treatment, metastasis, recurrence, and survival via phone, mail, and outpatient visits. Tumor recurrence, metastasis, the time of metastasis or recurrence, and the survival were recorded.

### 2.7. Statistical Analysis

Patients' characteristics were shown as mean ± SD for continuous variables and as counts and percentages for discrete variables. SPSS version 26.0 was used to analyze the data. The analysis methods consisted of the *t*-test, chi-square test, and Fisher's exact probability test. Statistical significance was set at *p* < 0.05.

## 3. Results

### 3.1. Clinical Features

All patients were followed up until May 2019, and complete data were obtained for the enrolled 51 patients with PGLs, 8 patients had PGLs with aggressive biological behavior, including 2 patients (3.9%) with local recurrence and 6 patients (9.6%) with metastasis (2, liver metastasis; 1, lung metastasis; 1, lung and bone metastasis; 1, liver and bone metastasis; and 1, liver, lung, and chest wall metastasis). The remaining 43 patients were generally in good condition and survived without recurrence or metastasis at the end of the follow-up period.

The details of the demographic and clinical characteristics have been summarized in Table 1. There were 30 female and 21 male participants; the mean ± SD age was 52.8 ± 14.5 (range, 21–77) years. Of the 51 patients, the majority (88.2%; 45/51) had nonspecific symptoms, and 6 were asymptomatic. The nonspecific symptoms included abdominal pain (20 participants, 39.2%), palpitations, discomfort when urinating, and dizziness. The difference of sex, age, clinical presentation, and abnormal endocrine were not significant between the two groups.

### 3.2. Analysis of CT Findings

Table 2 summarizes the CT findings of the PGLs. All 51 neoplastic lesions were solitary, and most of them had well-defined borders. The mean lesion size was 55.2 mm (mean diameter; range, 12–130 mm). The difference of the mean size between the nonaggressive PGLs group and the aggressive PGLs group was evident (*p* < 0.05). The mean diameter of the aggressive PGL lesions was 80.0 mm, while that of the nonaggressive PGLs lesions was 50.6 mm. The most common location was the retroperitoneum (*n* = 47, 92.2%), the lesions were distributed adjacent to the abdominal aorta or the inferior vena cava, and the surrounding organs were compressed towards the anterolateral side. In 3 (5.9%) patients, the tumors were located in the bladder. Other locations included the liver (1 patient) and the mesentery (1 patient) (Table 2).

Unenhanced CT scans presented that the mean density of the PGLs was 42 HU (range, 25–60 HU) and the mean contrast-enhanced CT value in the arterial phase was 97 HU (range, 45–185 HU) was 100 HU (range, 59–201 HU) in the venous phase (*n* = 32, 62.7%), respectively. In the aggressive PGLs group, most of them (*n* = 6, 75.0%) had more nonobvious CT enhancement degree (values <40 HU) than the nonaggressive PGLs group (*n* = 8, 18.6%). In the nonaggressive PGLs (*n* = 23, 53.5%), the majority of them had more significant CT enhancement degree (values >60 HU) than the aggressive PGLs (*n* = 1, 12.5%). CTA, CTV, DEAP, DEPP, and ED values were significantly different between the two groups. The differences in CTU and progressive enhancement or not had no significant difference between the two groups. The “pointed peach” sign was described as the tumor is embedded to the space of the adjacent organs and the blood vessels but without the vascular cavity and vascular wall invasion, which is another characteristic of PGLs.

**Figure 1:**
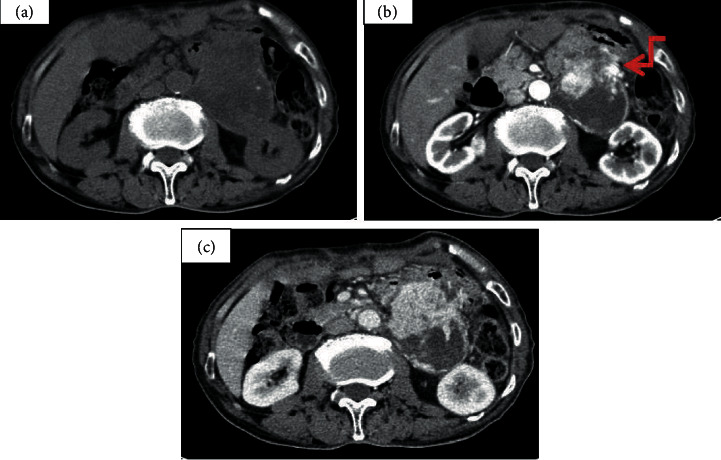
One case of a surgery confirmed retroperitoneal paraganglioma in a 77-year-old male that located in the left paraaortic area. (a) Nonenhanced CT scan presented a low heterogeneity density with an irregular shape tumour size of about 97 mm × 69 mm. The presence of multiple-size varied cystic degeneration and speckled calcification. (b, c) Dynamic enhanced CT scans revealed an obvious heterogeneously enhanced tumour for solid components. Peritumor and intratumor blood vessels (arrow). CT values were 32, 154, and 134 Hounsfield units (HU), respectively, in the nonenhanced, arterial, and venous phases.

**Figure 2:**
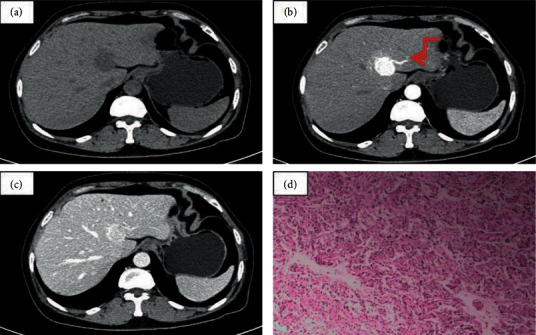
One case of a pathologically confirmed liver paraganglioma in a 60-year-old female. (a) Noncontrast CT scan showed a well-defined lesion in the liver segment IV with a mean diameter of 22 mm. (b, c) Contrast-enhanced CT scans showed homogenous and significant enhancement with the “halo sign,” peritumor feeding, or draining enlarged vessels (arrow). The CT values of the noncontrast, arterial, and venous phases were 35, 199, and 120 HU, respectively. The lesion was misdiagnosed as benign hemangioma or FNH tumour following preoperative CT. (d) The tumor cells were arranged in nests, surrounded by fibrovascular stroma.

**Figure 3:**
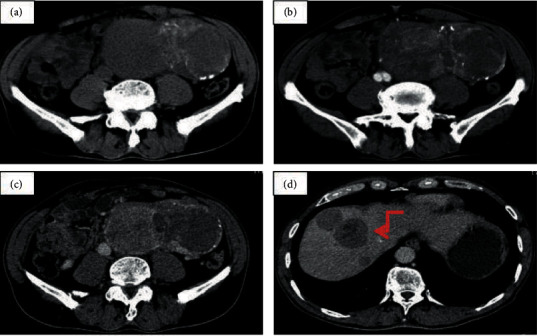
One case of a 65-year-old female that had a slightly higher-level CA199, CA125. (a) Noncontrast CT scan showed an irregular tumour size of about 140 × 81 mm with heterogeneous low density in the retroperitoneal. The lesion presence multiple punctate strip calcifications. (b, c) Contrast-enhanced CT scans depicted heterogeneous nonsignificant enhancement. The CT values of the noncontrast, arterial, and venous phases were 43, 67, and 65 HU. The postoperative CT diagnosis was paraganglioma and (d) liver multiple metastasis (arrow) occurred.

**Figure 4:**
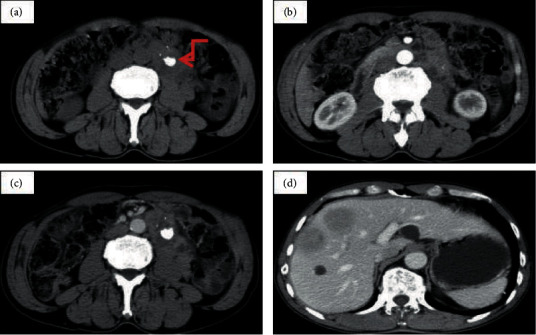
One case of a 67-year-old female with a retroperitoneal neoplasm. (a) Noncontrast CT scan showed an irregular, ill-defined tumour with heterogeneity attenuation and bulky calcification (arrow) was observed. (b, c) Contrast-enhanced scans displayed heterogeneous nonremarkable enhancement. The CT values of the noncontrast, arterial, and venous phases were 43, 79, and 88 HU. The neoplasm extends into the space of the adjacent organs and the left renal artery. (d) Liver multiple metastases occurred 5 years later.

In this current study, 67.4% (29 of 43) of the nonaggressive PGLs and 62.5% (5 of 8) of the aggressive PGLs displayed the “pointed peach” sign.  Figure 1 presents one case of a 77-year-old man with an irregular-shaped heterogeneous retroperitoneal tumor (size, 97 mm × 69 mm) showing an obvious inhomogeneous enhancement pattern. There was no significant difference in the field of morphology between the nonaggressive PGLs group and the aggressive PGLs group. All (8/8) of the aggressive PGLs group were irregular masses, while only 55.8% (24/43) of the nonaggressive PGLs group had irregular shape.  Figure 2 shows one case of a 60-year-old woman with a well-defined tumor in segment IV area of the liver. The big feeding and draining vessels crossed the lesion without invasion. It was misdiagnosed as a cavernous hemangioma or focal nodular hyperplasia. The majority of the aggressive PGLs (87.5%, 7/8) demonstrated enlarged peritumor and intratumor vessels, whereas roughly half of the nonaggressive PGLs (58.1%, 25/43) exhibited these vessels. The presence of enlarged peritumor and intratumor vessels feeding or draining the mass was not significantly different between the two groups.  Figure 3 demonstrates the CT examination of an old woman with an irregular tumor that had multiple punctate or nodular calcifications; then, liver metastasis happened four years later. The presence of calcification was not significantly different between the aggressive PGLs group and nonaggressive PGLs group.  Figure 4 presents one case of an old woman with an irregular, ill-defined retroperitoneal neoplasm with a bulky calcification. Liver metastasis occurred 5 years later. Among the entire cohort, eight patients had recurrences and metastases 4–10 years after excision in a mean follow-up period of 10 years.

## 4. Discussion

The PCC was defined as a neuroendocrine tumor originating from the adrenal medulla; meanwhile, PGLs arise from extra-adrenal paraganglia. The incidence of PCC and PGLs ranges between 2 and 8 per million. Extra-adrenal PGLs are rare, highly vascular, nonepithelial tumors arising from chromaffin cells in the ganglia of the autonomic nervous system and the accompanying nerves. They account for about 0.06% of all PGLs. Abdominal PGLs are mostly a single irregular mass in the organ of Zuckerkandl or bladder base [16–18]. These current results show the 51 cases were all solitary, and most of them were located around the retroperitoneal abdominal aorta, which was consistent with previous reports [19]. Previous literature [20, 21] reported an average age of 49.8 (16–76) years and with an equal incidence in male and female. The average age of our study population was 52.8 (range, 16–77) years, and the male-to-female ratio was 10 : 7, which was consistent with the previous literature generally. PGLs could be divided into functional and unfunctional types, depending on the catecholamine content and the degree of catecholamine release. In this current study, the clinical symptoms of unfunctional PGLs included headache, vomiting, nausea, weight loss, abdominal pain, and abdominal distension. Patients with functional PGLs can develop palpitations, dizziness, high blood pressure. The clinical manifestations for the diagnosis of PGLs are insufficient. A sufficient diagnosis of PGL depends on the typical pathological features of the tumor tissue. In the present study, the surgical procedures provided ample evidence for a sufficient pathological diagnosis.

Management of abdominal PGLs, including their diagnosis, is difficult because they have no characteristic symptoms and imaging features. As far as we know, the current study describes the larger sample size of abdominal PGLs on CT characteristics in detail. CT mainly demonstrated an isolated well-defined mixed-attenuation irregular nodule or mass with varying degrees of internal hemorrhage and cystic degeneration. The cysts included a single large cyst, a single small cyst, and multiple different-size cysts. In this group, most single-shot irregular similar soft tissue density or slightly lower inhomogeneous density was observed in PGLs on nonenhanced CT examination. High-density calcification lesions were shown in 11cases, mainly with a mottling, patchy, and linear-shaped scattered distribution. The previous reports show that the presence of calcification has been regarded as a representative feature for the diagnosis of PGLs [22, 23]. Pathologically, PGLs are classified as type I if the main components are lobules or nests of the chief cells and type II if they are surrounded by a single layer of sustentacular cells and sporadic dystrophic calcifications due to sustentacular cells.

Typically, dynamic-contrast CT examination showed that the apparent enhancement pattern of the lesion was helpful for the diagnosis of PGLs. The lesions usually have enlarged peritumor and intratumor vessels feeding or draining the mass [24]. They reflect the rich blood supply of the PGLs, which is consistent with the pathological characteristics of tumors composed of the main cells, support cells, and rich blood sinuses forming a vascular network. Large PGLs are susceptible to degeneration and tumors show necrosis, cystic changes, and hemorrhage. Our study demonstrated that some of the large tumor necrosis cysts were completely deformed and resembled the “island sign.”

PCC have a potential for malignancy and metastasis; their malignancy rate is 2–10%. PGLs have a higher frequency of metastasis than PCC. Retroperitoneal PGLs are more aggressive, metastasizing in up to 42% of cases [25]. There is no reliable method of diagnosing PGLs as malignant; therefore, the malignancy is usually evidenced by the presence of metastases (synchronous or heterochronic), recurrence, or local invasion [26]. However, patients with malignant PGLs have a low survival rate, without early identification of possible malignant tendencies, the long-term prognosis does not improve. Currently, there is great interest in developing new tools to predict and diagnose malignancy. This has prompted the search for other clinical features, tumor markers, and CT features that may correlate with malignant behavior. We evaluated the differences between several clinical features, tumor CT image features, and tumor markers of PGLs to predict the recurrence or metastasis of these lesions. In previous research [27], it was noticed that the aggressiveness in PGLs was more common in patients who present with a PGL measuring larger than 5 cm and/or an SDHB mutation carrier. In addition, tumor size can provide crucial risk information for the prognosis of PGLs. The current study showed that the tumor size was significantly different between the two groups. This was not consistent with a previous report; our cut-off point was 8 cm.

In the current study, there were no statistically significant differences in the presence or absence of cystic degeneration, calcification, hemorrhage, enlargement of lymph nodes, peritumor or intratumor blood vessels, and between the aggressive PGLs group and nonaggressive PGLs group. Therefore, the presence or absence of cystic changes, necrosis, calcification, and the “pointed peach” sign cannot be used as a criterion for distinguishing the benign or malignant nature. One possible explanation is that the tumor mean diameter between the two groups was larger than 5 cm and the occurrence rate of cystic degeneration, calcification, and hemorrhage may be size-related. In addition, owing to limited posterior peritoneal space, the “pointed peach” sign was more likely to occur and extends to adjacent organs when the lesion measures larger than 5 cm. In the present study, the CTA and CTV values of the aggressive PGLs were less than those of the nonaggressive PGLs; meanwhile, the DEAP and DEPP values were larger in the aggressive PGLs than in the nonaggressive PGLs. All differences had significant statistical significance between the two groups (*P* < 0.05). Compared to the nonaggressive PGLs, the aggressive PGLs were more likely to have a mild-to-moderate enhancement degree (<40 HU), whereas the nonaggressive PGLs were more likely to have a significant enhancement degree (>60 HU). The possible mechanism for these enhancement differences may be associated with the rapid growth of aggressive tumors and the relatively slow angiogenesis within the tumor. Another possible explanation is that aggressive PGLs are generally larger and more prone to cystic and necrosis. Most of them were more likely to have a progressive enhancement pattern (62.7%, 32/51) and there was no significant difference between the two groups; One possible explanation for this progressive enhancement pattern due to the contrast agent accumulation in the abundant sinusoidal dilated fibrovascular stroma between nests, and the contrast agent is slowly cleared.

There are still several limitations to the present study. Firstly, it was a retrospective observational study of imaging with a limited sample size (51 patients), which was a limitation owing to the risk of selection bias. Therefore, more polycentric prospective studies are necessary to assist these CT conclusions. Secondly, CT images were acquired by using three different types of CT scanners, which may have influence the consequences. The latter, due to lack of follow-up in the long term for the more recent cases, we did not have information on whether the patients had recurrence or died.

## 5. Conclusion

In summary, the current study describes the large series of abdominal PGLs about CT features in detail and attempts to predict malignancy. The tumor that is larger than 8 cm and nonsignificant enhancement should be regarded as high-risk factors for recurrence or malignancy. These factors should be recommended as a new approach for appropriate clinical management and strict follow-up.

Metastases may be microscopic at the time of initial surgery. In our series, metastasis may have become evident 10 years after surgical removal of the primary tumor. Long-term follow-up for over 10 years is necessary for patients with benign PGLs at yearly intervals. However, the PGL patients with high risk for having a large tumor diameter over 8 cm or/and nonsignificant enhancement should be followed up every six months and reference intervals throughout life.

## Figures and Tables

**Table 1:** Demographic and clinical characteristics of the 51 cases of paragangliomas.

	All patients	The aggressive group	Nonaggressive group	*p* value
Number of patients	51	8	43	/
Age ± SD	52.8 ± 14.5	50.5 ± 11.4	53.3 ± 15.1	0.622
Male/female ratio	30/21	6/2	24/19	0.272
Symptoms				
Abdominal pain	20 (39.2%)	4 (50%)	16 (37.2%)	/
Dysuria	3 (5.9%)	0 (0.0%)	3 (7.0%)	/
Health check-up	6 (11.8%)	1 (12.5%)	5 (11.6%)	/
*Endocrine examination*				
Cortisol	2 (3.9%)	0 (0.0%)	2 (4.7%)	1.000
CA199	3 (5.9%)	1 (12.5%)	2 (4.7%)	0.407
CEA	4 (7.8%)	0 (0.0%)	4 (9.3%)	1.000
CA125	3 (5.9%)	2 (25%)	2 (4.7%)	0.111
Renin	2 (3.9%)	0 (0.0%)	2 (4.7%)	1.000

Data were presented as mean SD or *n* of patients (%). There were no significant between-group differences (*p* > 0.05); continuous data were compared using an independent two-sample *t*-test or the Mann–Whitney *U*-test based on data normality; categorical data were compared using Fisher's exact test. Note: CA199 = carbohydrate antigen 199; CEA = carcinoma embryonic antigen; CA125 = glycogen antigen 125.

**Table 2:** CT findings of the 51 cases of paragangliomas.

	All patients	The aggressive group	Nonaggressive group	*p* value
Number of patients	51	8	43	/
CT values (HU)				
CTU	42	43	42	0.595
CTA	97	71	102	0.020 ^*∗*^
CTV	100	80	104	0.020 ^*∗*^
DEAP	55	60	6	0.020 ^*∗*^
DEPP	58	62	14	0.023 ^*∗*^
ED				0.009 ^*∗*^
Enhancement degree (<40 HU)	14 (27.4%)	6 (75.0%)	8 (18.6%)	
Enhancement degree (>40 HU, <60 HU)	13 (25.5%)	1 (12.5%)	12 (27.9%)	
Enhancement degree (>60 HU)	24 (47.1%)	1 (12.5%)	23 (53.5%)	
EP	2.5	1.9	2.6	
Progressive enhancement	32 (62.7%)	7 (87.5%)	25 (58.1%)	0.238
LD (mm)	61.9	89.8	56.7	0.008 ^*∗*^
SD (mm)	48.5	70.2	44.4	0.007 ^*∗*^
Mean D (mm)	55.2	80	50.6	0.007 ^*∗*^
LD/SD	1.29	1.33	1.29	0.768
Location				0.580
Retroperitoneal	47 (92.2%)	8 (100.0%)	38 (88.4%)	
Else	5 (9.8%)	0 (0.0%)	5 (11.6%)	
Morphology				0.057
Round	4 (7.8%)	0 (0.0%)	4 (9.3%)	
Oval	15 (29.4%)	0 (0.0%)	15 (34.9%)	
Irregular	32 (62.8%)	8 (100.0%)	24 (55.8%)	
Solid	6 (11.8%)	0 (0.0%)	6 (14.0%)	0.471
Cystic degeneration	45 (88.2%)	8 (100.0%)	37 (86.1%)	0.640
Single and large	3 (5.9%)	1 (12.5%)	2 (4.7%)	
Singe and small	12 (23.5%)	1 (12.5%)	11 (25.6%)	
Multiple	30 (58.8%)	6 (75.0%)	24 (55.8%)	
Hemorrhage	1 (2.0%)	1 (12.5%)	0 (0.0%)	0.052
Calcification	11 (21.6%)	8 (18.6%)	3 (37.5%)	0.468
“Pointed peach” sign	34 (66.7%)	5 (62.5%)	29 (67.4%)	1.000
Lymph nodes	3 (5.9%)	1 (12.5%)	2 (4.7%)	0.407
Blood vessels				0.577
None	5 (9.8%)	0 (0.0%)	5 (11.6%)	
Intratumoral	7 (13.7%)	0 (0.0%)	7 (16.3%)	
Peripheral	7 (13.7%)	0 (0.0%)	7 (16.3%)	
All	32 (62.8%)	7 (87.5%)	25 (58.1%)	

Data are presented as *n* of patients (%). Continuous data were compared using an independent two-sample *t*-test or the Mann–Whitney *U*-test based on data normality; categorical data were compared using Fisher's exact test. Values written with “ ^*∗*^” indicate a significant difference between the tumors (*P* < 0.05). Note: CTU = CT value of nonenhancement; CTA = CT value of arterial phase; CTV = CT value of venous phase; DEAP = degree of enhancement in arterial phase; DEPP = degree of enhancement in portal venous phase; ED = enhancement degree; EP = enhancement potentiality; LD = long diameter; SD = short diameter.

## Data Availability

The data used to support the findings of this study are available from the corresponding author upon request.
